# Effectiveness and safety of tacrolimus with or without eltrombopag, as a part of immunosuppressive treatment of aplastic anemia in adults: a retrospective case series

**DOI:** 10.1007/s00277-021-04401-6

**Published:** 2021-01-09

**Authors:** Anastasia Martynova, Victor Chiu, Melissa Mert, David Hermel, Ilene Ceil Weitz

**Affiliations:** Jane Anne Nohl Division of Hematology, Keck-USC School of Medicine, Los Angeles, CA USA

**Keywords:** Aplastic anemia, Immunosuppressive therapy, Tacrolimus, Eltrombopag, Cyclosporine

## Abstract

First-line treatment of aplastic anemia(AA) and for AA patients ineligible for hematopoietic stem cell transplantation (HSCT) has consisted of antithymocyte globulin (ATG), the calcineurin inhibitor cyclosporine A (CsA), and more recently eltrombopag. However, at our institution, we have successfully substituted another calcineurin inhibitor, tacrolimus, as a part of immunosuppressive threatment (IST) for AA due to more favorable toxicity profile. Since there is limited data on the use of tacrolimus in aplastic anemia, we conducted a retrospective review of twenty patients treated with tacrolimus-based immunosuppressive therapy (IST) as a first- or second-line treatment. The overall response rate was comparable to that of patients treated with CsA (18 patients). However, there were no cutaneous side effects observed in patients receiving tacrolimus, a relatively common finding with CsA use. Our data suggest that tacrolimus-based IST is a potential option in AA and might have a more favorable toxicity profile compared to CsA.

## Introduction

Immunosuppressive therapy (IST) is the cornerstone of acquired aplastic anemia treatment in adults who are of advanced age or do not have an allogeneic bone marrow transplant (BMT) donor available. Treatment with immunosuppressive therapy (IST) using the combination of anti-thymocyte globulin (ATG) and cyclosporine A (CsA), with the more recent addition of thrombopoietin receptor (TPO) agonist, eltrombopag, has been shown to be an effective and safe first-line regimen [1, 2]. Published response rates range from 50 to 70%, with the majority of patients achieving long-term survival [3, 4]. While many second-line therapies have been recommended, outside of hematopoetic stem cell transplantation (HSCT), there is limited data on the efficacy and safety of therapies for adult patients who have failed to respond to CsA or developed CsA-associated toxicities [4, 5].

Cyclosporine and tacrolimus are widely used in post-allogenic bone marrow as well as in solid organ transplant patients to prevent graft vs host or acute rejection [6–11]. The mechanism of action of both drugs is the suppression of T cell activation via downregulation of calcineurin, leading to decreased intranuclear translocation of nuclear factor of activated T cells (NFAT). Tacrolimus is a macrolide antibiotic that binds FK-binding protein, while cyclosporine is a lipophilic cyclic eleven amino acid peptide that binds to cyclophilins [12]. As an immunosuppressive agent, tacrolimus is approximately 100 times more potent than cyclosporine [13]. There are case series of successful use of tacrolimus as part of an immunosuppressive therapy regimen in pediatric patients with aplastic anemia [14, 15]. However, IST using tacrolimus has not been well described in adult patients with AA. To our knowledge, only one study has been published describing the use of tacrolimus for aplastic anemia in adults [16]. Zhu et al. used rabbit anti-thymocyte globulin (ATG) instead of horse ATG, which is more often used in the first-line setting in the USA [2, 17]. There is no data using tacrolimus and eltrombopag.

Differences in toxicity profiles between the two agents have been described in the solid organ transplantation literature and suggest that tacrolimus may be an attractive treatment alternative to cyclosporine. Toxicities associated with cyclosporine such as acute kidney injury, hypertension, neuropsychiatric symptoms, and cutaneous side effects such as gum hypertrophy and hirsutism are common, especially in women [18, 19]. Tacrolimus causes less cutaneous toxicities in renal transplant patients, and is associated with less calcineurin inhibitor-associated nephrotoxicity [20, 21]. Patients receiving tacrolimus after lung transplant have lower incidence of bronchiolitis obliterans and hypertension [9]. A systemic review also showed less hypertension, hyperlipidemia, and cutaneous toxicities in patients who received tacrolimus after heart transplant compared to those that received cyclosporine [7].

In this study, we restrospectively examined the safety profile and effectiveness of tacrolimus combined with ATG with and without eltrombopag, in both first-line and second-line immunosuppressive therapy in patients at an large urban academic medical center.

## Methods

This study was a retrospective case series conducted at the University of Southern California Norris Comprehensive Cancer Center and LAC+USC Medical Center. Using ICD-9 codes, we searched the administrative databases of these two large hospital systems serving a diverse, urban population. Patients with new or established diagnosis of aplastic anemia (AA) who received either cyclosporine (CsA), tacrolimus, or both as part of an immunosuppressive regimen from 2008 to 2017 were included in this series. We abstracted baseline demographic and laboratory data for these patients, as well as details regarding immunosuppression, hematologic response, and toxicities from treatment. We used the Camitta criteria to classify severity of aplastic anemia as moderate, severe, and very severe [22]. Response was evaluated at 3, 6, 9, and 12 months after therapy initiation. Complete response (CR) was defined as hemoglobin (Hgb) > 10 g/dL, absolute neutrophil count (ANC) > 1000, and a platelet count > 100,000 cells/mcL maintained for at least 3 months. Partial response (PR) for patients starting with very severe or severe aplastic anemia was defined as achieving hematologic indices that did not meet criteria for CR, but no longer met Camitta criteria for severe aplastic anemia. PR for patients starting with moderate aplastic anemia was defined as transfusion independence and an improvement in at least one cell line as follows: Hgb increase by 3 g/dL, ANC by 500 cells/mcL, and platelet count improvement by 20,000 cells/mcL. Relapse was defined as declining blood counts no longer meeting criteria for PR. The overall response rate corresponded to the proportion of patients who had a partial or complete response. Acute kidney injury (AKI) was defined as increase in creatinine > 1.5 mg/dL times baseline.

### Compliance with ethical standards

This study was approved by the University of Southern California Institutional Review Board (USC IRB) in accordance with the World Medical Association’s Declaration of Helsinki. Because this was a retrospective chart review and patients were de-identified, informed consent was not required by the USC IRB. No relevant conflict of interest was identified for the authors ICW, AM, VC, MM, and DH.

This paper was funded by a gift from the Tilley Famiy Foundation.

### Statistical analysis

Categorical variables are reported as absolute number and percentage; continuous variables are reported as median (interquartile range [IQR]). Differences in demographics and baseline characteristics by first line of treatment were tested by Fisher’s exact test or exact Wilcoxon rank sum test, as appropriate. Differences in treatment response at 3, 6, and 12 months were tested by Fisher’s exact test. Statistical significance was set at *p* < 0.05. All statistical analyses were performed using SAS 9.4.

## Results

We identified 36 patients with aplastic anemia who were treated with either CsA, tacrolimus, or both. Six patients were excluded from the final analysis due to lost of follow-up within 2 months of presentation. Of the remaining 30 patients, 20 patients received CsA + ATG and 10 patients received tacrolimus + ATG IST as first-line treatment. All but one patient received horse ATG as a part of IST and the remaining one patient in CsA group received rabbit ATG. Three patients proceeded with HSCT within 1 year of IST initiation. Out of those 3, one patient had moderate AA, one severe AA, and one very severe AA. Patients with severe and very severe AA received CsA + ATG as initial IST after diagnosis. One patient had only partial response and proceeded his treatment with haploidentical BMT. The other patient was nonresponder and had salvage therapy with allogenic stem cell transplant. The remaining patient with moderate AA initially that received tacrolimus + eltrombopag was nonresponder and proceed with HSCT.

There were 18 females and 12 males with a median (IQR) age of 46 (32–67) years. Fifty percent of the study patients were Latino. Tacrolimus was initialted at 3 mg twice daily. Of all patients who received tacrolimus as either line therapy (21), twelve received eltrombopag as a part of IST. Eltrombopage was initialed at 150 mg daily in Caucasian and African American patients, 75 mg daily in Asian patients. In all but one patient, eltrombopag was started within 3 weeks of IST initiation (median time 6 days). The remaining one patient was started 2 months after IST initiation due to insurance issues.

Patients taking tacrolimus vs. cyclosporine as initial therapy did not differ in terms of demographics, although patients receiving first-line tacrolimus were significantly more likely to be concurrently taking eltrombopag compared to patients taking cyclosporine (81.8% vs. 36.8% respectively; *p* = 0.01; Table 1). This is due to majority of patients in tacrolimus cohort being diagnosed and treated after eltrombopag was reported to improve hematopoiesis in severe AA [23].

**Table 1:** Demographics and patient characteristics by first line of treatment (*n* = 30)

Characteristic	Total (*n* = 30)	Cyclosporine (*n* = 20)	Tacrolimus (*n* = 10)	*p* value
Age, *years*	46 (32–67)	43 (35–66)	48 (32–69)	0.86
Gender				
Female	18 (60.0%)	11 (52.6%)	7 (72.7%)	0.41
Male	12 (40.0%)	9 (47.3%)	3 (27.2%)	
Ethnicity				
Asian	5 (16.6%)	4 (21.0%)	1 (9.0%)	0.66
Hispanic	15 (50%)	10 (47.3%)	5 (54.5%)	
White	10 (33.3%)	6 (31.5%)	4 (36.3%)	
Severity *				
Moderate	10 (33.3%)	7 (36.8%)	3 (27.2%)	0.25
Severe	9 (30%)	4 (15.7%)	5 (54.5%)	
Very severe	6 (20%)	4 (21.0%)	2 (18.1%)	
BM cellularity, *%*	10 (5–10)	10 (5–10)	10 (5–10)	0.66
Horse antitimocyte globulin	27 (90%)	17 (84.2%)	9 (90.9%)	> 0.99
Rabbit antitimocyte globulin	3 (10%)	3 (15.7%)	0	0.53
Eltrombopag use	17 (56.6%)	8 (36.8%)	8 (81.8%)	0.01
Length of follow-up (days)	955.0 (768.0–1739.0)	1159.5 (832.0–2264.0)	708 (438–780)	–

Results are reported as median (IQR) or *N* (%), as appropriate

*5 missing observations in cyclosporine group

The response and duration of tacrolimus therapy were described in Table 2. Toxicities of any kind were not statistically significantly different by first-line therapy (Table 3). All seven patients that stopped cyclosporine therapy due to toxicity (three with gingival hyperplasia, three with hirsutism, one with transaminitis) were switched to tacrolimus maintanence therapy and experienced resolution of the above side effects within 3 months after CsA discontinuation.

**Table 2:** Response to tacrolimus-based IST in patients with AA

Pt#	Age (years), gender	Ethnicity	Severity of AA	Clinical PNH	Eltrombopag use	Type of ATG	Time to TI (days)	Response at 3 months	Response at 6 months	Response at 12 months	Duration of tacrolimus treatment (days)	Time to relapse (days)	Length of follow-up (days)	Tacrolimus side effects
1*	27, F	Asian	Very severe	No	Yes	Horse	29	PR	CR	CR	404		404	
2*	69, F	White	Moderate	No	Yes	Horse	N/A	PR	PR	PR	1031		1031	AKI
3*	48, M	Hispanic	Moderate	No	Yes	Horse	79	NR	PR	PR	438		438	
4*	42, F	Hispanic	Severe	No	No	Horse	191	PR	PR	PR	337	487	768	AKI
5*	32, M	Hispanic	Severe	No	Yes	Horse	37	PR	PR	PR	817	416	1014	
6*	23, F	Hispanic	Severe	No	Yes	Horse	114	PR	PR	PR	784		486	
7*	71, F	White	Severe	No	No	Horse	N/A	PR	PR		106		106	
8*	73, F	White	Severe	No	Yes	Horse	18	CR	CR	CR	780	416	780	
9*	52, F	White	Very severe	No	Yes	Horse	113	NR	PR	PR	708	364	708	AKI
10*^+^	45, M	Hispanic	Moderate	No	Yes	Horse	N/A	NR	NR	N/A	268		309	
11	26, F	Asian	Very severe	No	No	Horse	N/A	PR	CR	CR	1151		1335	
12	35, F	Hispanic	Moderate	No	Yes	Horse	0	CR	CR	CR	465		912	
13	25, M	Hispanic	Moderate	No	No	Horse	7	NR	PR	CR	968		968	AKI
14	26, M	White	Severe	No	No	Horse	13	PR	PR	CR	1760		1971	Gynecomastia
15	66, M	White	Moderate	No	Yes	Horse	N/A	NR	NR	NR	809		809	
16	38, F	White	Moderate	Yes	No	Horse	N/A	NR	NR	NR	775		775	
17	70, F	Hispanic	Moderate	Yes	No	Horse	84	NR	NR	NR	832		832	
18	35, F	White	Very severe	No	Yes	Horse	N/A	PR	PR	NR	250		984	Diarrhea
19	35, F	Hispanic	Very severe	No	Yes	Horse	N/A	NR	NR	NR	795		955	Transaminitis
20	29, F	Asian	Moderate	Yes	No	Rabbit	N/A	NR	NR	NR	578		826	AKI
21	47, M	Hispanic	Severe	No	No	Horse	N/A	NR	PR	PR	757		757	Transaminitis

*First-line tacrolimus-based immunosuppressive therapy

+ Patient received HSCT during the treatment course

Top 10 patients received tacrolimus + ATG as a first-line IST at the time of diagnosis. The remainder 11 patients were switched to tacrolimus maintenance therapy due to poor response to Cyclosporin A (13, 15, 18, 20) or intolerable toxicities (11, 12, 14, 16, 17, 19, 21). Of patients switched due to toxicities, patient #10 was in PR on CsA at the time of switching, and all other patients did not achieve desirable response on CsA; however, the primary reason to switch medication was development of toxicity which in most cases was AKI. At the time of the last review, 4 patients (8, 12, 14, 21) were weaned off tacrolimus and continued to remain in remission, 12 patients remained on tacrolimus therapy, 3 stopped tacrolimus due to adverse events, and 1 patient was lost of follow-up. Of 21, 3 patients (5, 8, 9) experienced relapse while on tacrolimus therapy and received second induction therapy with ATG + tacrolimus with response. One patient in this cohort (10) proceeded with HSCT (*Pt* patient; *AA* aplastic anemia; *PNH* paroxysmal nocturnal hemoglobinuria; *ATG* anti-thymocyte globulin; *TI* transfusion independence; *AKI* acute kidney injury; *HSCT* hematopoietic stem cell transplantation; *PR* partial response; *CR* complete response; *NR* no response; *N/A* not applicable)

**Table 3:** Odds of toxicity for tacrolimus vs. cyclosporine (*n* = 30)

	First-line therapy only (*n* = 30)		Patients switch from CsA to TAC (*n* = 11)	
Toxicity	Cyclosporine (*n* = 20)	Tacrolimus (*n* = 10)	Cyclosporine	Tacrolimus
Acute kidney injury	7 (38.9%)	3 (33.3%)	2 (18.2%)	2 (18.2%)
Skin	1 (5.6%)	0	1 (9.1%)	0
Hirsutism	3 (16.7%)	0	3 (27.3%)	0
Gum hyperplasia	3 (16.7%)	0	2 (18.2%)	0
Other*	6 (33.3%)	0	4 (36.4%)	4 (36.4%)

Results are reported as *N* (%)

^a^Firth’s odds ratios with profile penalized likelihood confidence limits and *p*-values of tacrolimus vs. cyclosporine treatment groups are reported. Models are adjusted for differences in follow-up time

*Other toxicities include hypertension (1), skin rash (1), transaminitis (2), nausea (1), tremors (1)

In six patients treated with tacrolimus, medication was held secondary to toxicity (4 AKI, 2 transaminitis, Table 3). Five of six patients were able to restart tacrolimus at the lower dose upon resolution of the adverse event and one patient was started on CsA as an alternative therapy. None of the toxicities recurred with subsequent treatment. The odds of experiencing acute kidney injury for patients on tacrolimus compared to CsA was not statistically significant (*p* = 0.54).

Patients who received tacrolimus as a second-line IST (*n* = 11) did not experience any mucocutaneous side effects, hirsutism, or gum hyperplasia that they had experience with their first-line cyclosporine treatment (Table 3). The median (IQR) change in the number of toxicities was 0 (− 1–0), which was not statistically significantly difference from 0 based on the Wilcoxon signed rank test (*p* = 0.13). In the ptients receiving tacrolimus, there were no additional toxicities associated with the addition of eltrombopag.

## Discussion

HSCT remains a first-line treatment option for patients diagnosed with severe AA but may not be feasible for some patients available. Our study primarily consists of patients from financially disadvantaged population who were not able to proceed with HSCT due to lack of insurance or absence of matched sibling donor. Our case series supports the use of tacrolimus as a viable treatment option for adult patients with AA. As we reported previously, tacrolimus showed durable response in majority of patients in both first-line and second-line treatment groups [24]. Although CsA had previously been the standard treatment option, in our institution, tacrolimus + ATG has been employed as a first-line IST as it showed better tolerability in the individual cases. The choice of tacrolimus therapy was mostly physician and patient driven due to unwanted cutaneous side effects to CsA leading to poor compliance.

Tacrolimus was well tolerated, and all patients remained on medication beyond 12 months. Two patients were lost of follow-up. Although the number of patients treated with tacrolimus was limited, our results did not show a difference in response with use of tacrolimus compared with CsA. The overall response rate was similar to that seen with ATG/CSA, but relapse was observed more frequently in the CsA group compared to tacrolimus group, 39% vs 15%, during a long period of follow-up.

Our findings are comparable to other studies examining first-line tacrolimus-based IST. In a study in pediatric population by Alsultan et al, an 88% complete response rate to tacrolimus + ATG as first-line IST was observed compared to 85% for CsA + ATG [14]. Similarly, the study by Zhu et al. reported an 85% OR rate and 54% CR rate in the tacrolimus group. In our series, 33% of patients achieved CR with tacrolimus and 55% with CsA at 12 months [16].

Observed toxicities in both groups were similar. Nephrotoxicity was the most common and clinically significant adverse effect of both groups although all the patients experienced resolution of AKI following medication cessation. AKI recurrence seemed to be similar in rate as well, suggesting that switching from CsA to tacrolimus due to AKI may not be not warranted. However, as expected, there was a clear increase in hirsutism and gingival hyperplasia with CsA. As aplastic anemia tends to effect younger age patients, these cosmetic side effects may lead to reduced therapy compliance and limit its overall use. Notably, in the CsA cohort, hirsutism was the reason of CsA discontinuation in 3 patients, two of whom were young females and one male.

Our study also describes the use and safety of concurrent administration of the TPO-agonist eltrombopag and tacrolimus. Eltrombopag plus standard IST using horse ATG and CsA has been demonstrated to improve blood counts and efficacy of the IST in the setting of refractory aplastic anemia. However it’s use in combination with tacrolimus has never been described [1, 25, 26]. In our study, most of the patients treated with any line tacrolimus received concurrent eltrombopag. It was well tolerated without an increase in toxicity. Treatment with eltrombopag has been reported to be associated with liver toxicity, which is a side effect of tacrolimus as well. In our cohort, only two patients treated with tacrolimus developed grade 3 elevation of liver enzymes. Only one received the tacrolimus and eltrombopag combination. Other patients who received both medications concurrently did not experience any hepatic toxicity, including transaminitis of any grade or other side effects. The combination appears to be effective and safe.

This is a relatively large case series of ethnically diverse patients with a rare hematologic disease for which there is limited data on treatment options. Our data set is unique in that half of our patients were from Latin America, where the incidence of AA is relatively low [27]. The majority of the patients in our cohort were patients in a resource-poor setting where bone marrow transplantation was not an option due to financial constraints. While these data show a favorable response rate to first-line tacrolimus, perhaps the most striking observation is that a large proportion of patients who do not tolerate CsA or who had not achieved a CR on CsA did achieve improved responses on tacrolimus. Notably, the median relapse-free survival for CsA users should be interpreted with caution as 27% of patients who failed first-line CsA and then proceeded with second-line tacrolimus have not been evaluated for response to CsA due to lack of data.

Our study is limited by its retrospective, uncontrolled design, small sample size, and different follow-up times for toxicity assessments. Further study with a prospective trial examining the use of tacrolimus with eltrombopag as first-line and later-line IST for aplastic anemia is warrented. However, in the absence of a prospective, randomized comparison, these data support the use of tacrolimus and eltrombopag as part of IST for AA.
